# Supplementary material for: Cultural linkage: the influence of package transmission on cultural dynamics
Source: Proc Biol Sci. 2019 Dec 4;286(1916):20191951. doi: 10.1098/rspb.2019.1951 (PMC6939269; doi:10.1098/rspb.2019.1951)

Appendix

S1. A model with non-transmitted links between cultural traits

To further understand whether the effects we describe in the main text come from the presence of links or as a result of the transmission of links, we ran a modified version of the simulation in which links between cultural traits are static and not transmitted. A model like this may correspond to world-views that vary between individuals but within individuals are constant relative to the rate of cultural transmission. Here, individuals associate certain traits even when they update some variants of those traits.

The overall design of this model is identical to the one described in the main text (see methods section for details) except links between cultural traits are not transmitted, as they are in the main text model. Specifically, after focal individuals chose their interaction partners and determined the packages for transmission, only the variants of the traits in the packages are transmitted and the links between traits in both the focal individual and the interaction partner remain unchanged. As a result, the parameter *b* no longer exists in the model and the parameter *a* (link formation) is set to zero so that no new links between traits are created during the simulation. For the initial condition, instead of starting with no links between traits, we randomly initialize the links, with each possible link existing with probability $l \in \{0, 0.1, 0.2, \ldots, 1\}$. We run simulations that investigate both pairwise difference in a population as a result of links between traits and cultural hitchhiking. Where hitchhiking is examined, a burn in period is no longer needed since the links are static. Instead, the adjustment described in the main text (under *Simulation set-up*) that leads to the most challenging trait distribution for hitchhiking now become the starting condition. That is, one innovator has trait 1 variant 1 (the most beneficial) and trait 2 variant 4 (the rarest), while all other individuals have trait 1 variant 4 (the most detrimental).

With this model we observe some of the effects of links on pairwise difference, that we see in the model with transmissible links (Fig. A1.1). As before, when there are no links or when the links are rare we get $\pi_1 > \pi^{WF}$ and when links are common we see that $\pi_1 = \pi^{WF}$ (Fig. A1.2). As described in

the main text, this result exists because links change the number of traits being transmitted per timestep. As the average package size increases, more transmission occurs, leading to lower pairwise difference.

However, unlike the model in which links between traits are transmitted, when the links are static at an intermediate frequency, π_1 is not less than π^{WF} (Fig. A1.2). Instead, the level of pairwise difference as a function of link frequency matches closely with the levels obtained in the original model with *b*=0.5. This occurs because where the configuration of large packages is not transmitted (or transmitted indeed at very low fidelity as is the case when *b*=0.5), the probability that a variant in a large package will remain in a large package after transmission is no higher than that probability for variants in small packages. In other words, the advantage of being in a large package is unlikely to persist over multiple timesteps.

We then compare the distributions of pairwise difference with (i) static links between traits and *l*=0.3, (ii) no links between traits, and (iii) the Wright-Fisher model. The distributions are shown in Fig. A1.3. The area of overlap between the static links model and the Wright-Fisher model is 0.87, and between the static links and no links model is 0.45. Therefore, the risk of falsely rejecting neutral transmission as the underlying mechanism still exists when links are not transmitted as with transmissible links.

Hitchhiking still occurs with static links, and again with more links (higher *l*), the more likely the associated variant is to reach a high frequency (Fig. A1.4).

Figure A1.1: Median pairwise difference under different package sizes. (A) with randomly selected traits in a package (same as Fig. 3A). (B) with static links.. The error bars indicate a 90% quantiles. Each point represents average across 100 simulations.

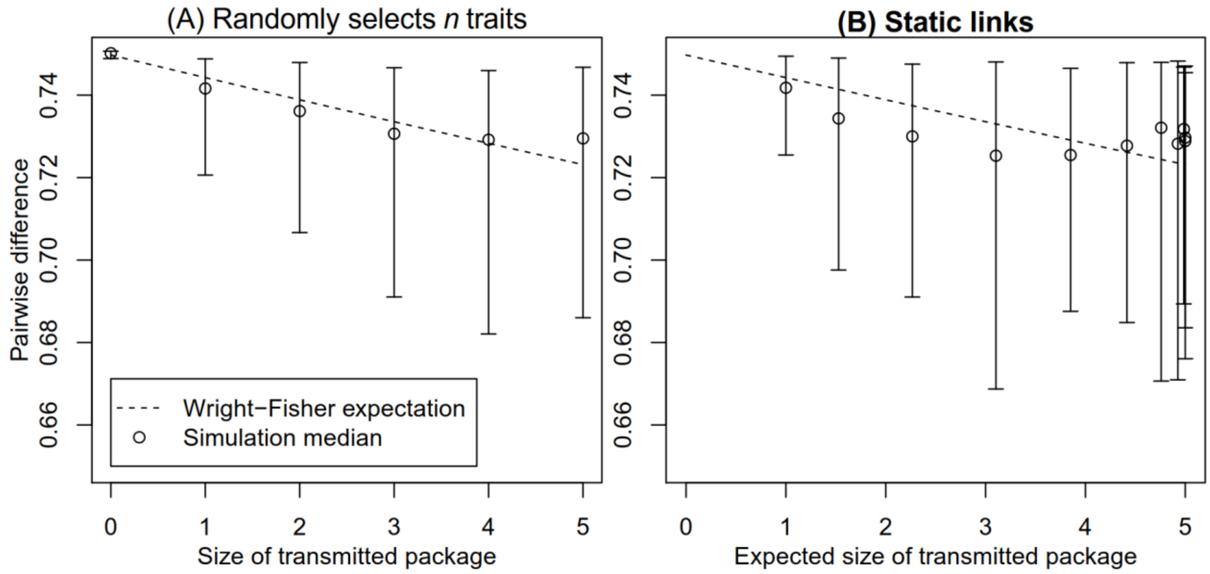

Figure A1.2: Mean pairwise difference under different link frequencies. To compare the results of the original model with the results of the static links model, we plot the frequency of links against the population pairwise difference (instead of parameter *a* against pairwise difference as in Fig. 2B) for both models. As before, when there are no links or when the links are rare we get $\pi_1 > \pi^{WF}$ and when links are common we get $\pi_1 = \pi^{WF}$ Here, with static links or with transmissible links with b=0.5, π_1 is not less than π^{WF} (see above). Each point in the static link model represents an average of 100 simulations.

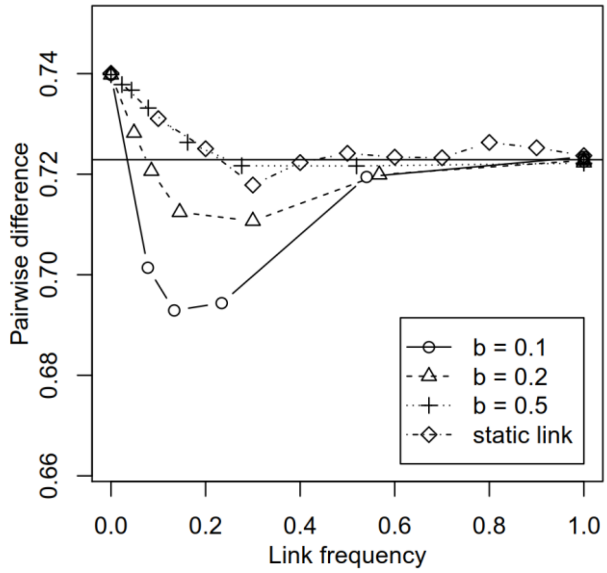

Figure A1.3: The distributions of pairwise difference under different linkage assumptions. For static links, l=0.3. The areas of overlap can be calculated (see above).

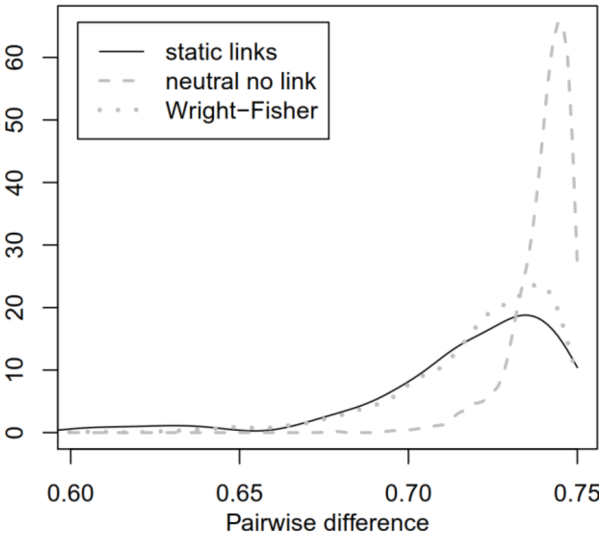

Figure A1.4: The hitchhiking of an associated variant. (A) Proportion of simulations in which a detrimental associated variant can reach a frequency of 0.5. (B) The number of timesteps the associated variant can remain at a frequency above 0.5 (calculated only if hitchhiking occurred in more than 10% of the simulations). Just like the case with transmissible links, the probability of hitchhiking occurring correlates positively with link frequency. In both measurements of hitchhiking, the results from non-transmissible link are similar to those from *b*=0.5. (s_1=0.9; s_2=0.2)

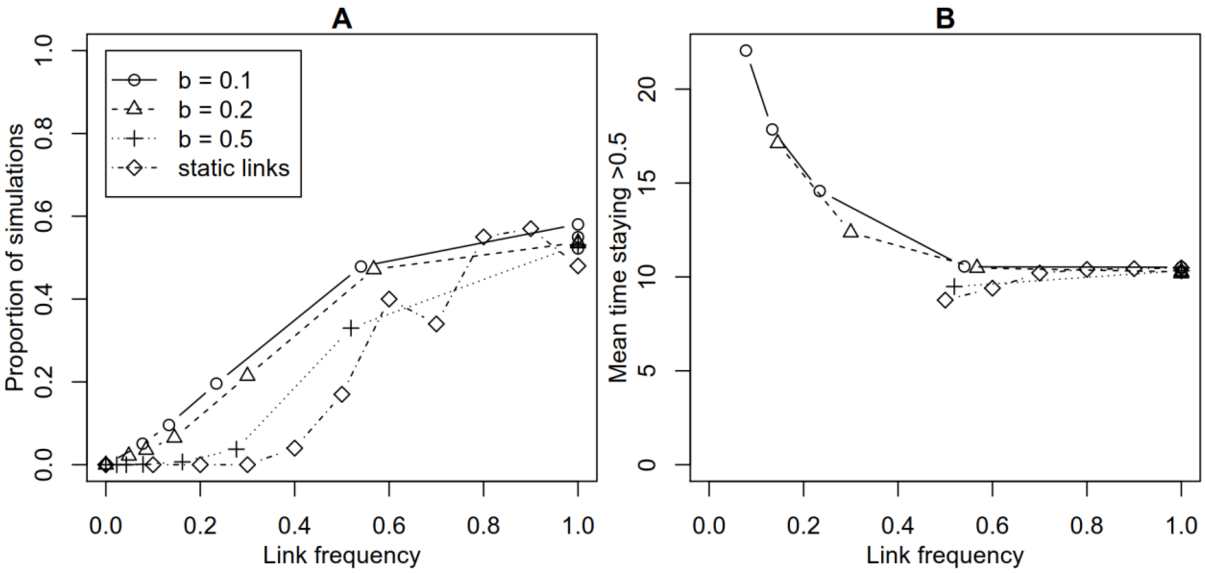

S2. Table A2: Parameters and their values used in the simulation.

Variable description	Symbols	Values
Rate of association (forming new links)	a	{0, 0.005, 0.01, 0.02, 0.05, 0.1, 0.2, 0.5}
Rate of breaking a link during transmission	b	{0.1, 0.2, 0.5}
Copy rate for each trait	c	0.99
Maximal payoff difference	s_1<br><br>s_2	$s_1 = 0.9$<br><br>$s_2 \in \{0, 0.1, 0.2\}$
Innovation rate	μ	0.01
Probability of being chosen as an interaction partner	p^r	Neutral:<br><br>$p^r = 1/(N\text{-}1)$<br><br>Payoff bias:<br><br>$p^r = \frac{\prod_{i=1}^{h}\left(1-\frac{j_{i,r}-1}{k-1}s_i\right)}{\sum_r \prod_{i=1}^{h}\left(1-\frac{j_{i,r}-1}{k-1}s_i\right)}$<br><br>Conformist bias:<br><br>$p^r = \frac{1-(1-q_r)s_\kappa}{\sum_r 1-(1-q_r)s_\kappa}$
Population size	N	1000
Number of traits	h	5 in neutral or payoff bias. 1 in conformist bias.
Number of variants per traits	k	4

S3. Robustness checks

To see whether our results are robust to changes in parameter values, we ran the simulation with the following changes in parameter values: a different copy rate, (*c*=0.8), a different innovation rate (μ=0.001), a different number of traits (*h*=8), a different number of variants (*k*=9), a different population size (*N*=200), varying *N, h*, and *k* simultaneously (*N*=200, *h*=8, *k*=2, or *N*=500, *h*=9, *k*=16. Only under neutral transmission).

In all parameter constellations that we investigated, the effect of links on pairwise difference follows the same qualitative pattern as described in the main text, with the exception of *c*, the copy rate. When there are no links between traits or when links are rare we find that $\pi_1>\pi^{WF}$, when links are common we see that $\pi_1=\pi^{WF}$, and when the links are at an intermediate frequency and *b* is small, we see that $\pi_1<\pi^{WF}$ (Fig. A3.1). Lowering *c* leads to higher pairwise difference because less transmission occurs (Fig. A3.1C).

We also tested the robustness of our claims regarding the equifinality of neutral and linked models to changes in parameter values. The area of overlap between the distributions of pairwise difference under three different linkage assumptions: "linked" *a*=0.01 *b*=0.1, "Wright-Fisher", and "unlinked" a=0) can again be calculated. In general, the relative positions of the distributions remain similar (Fig. A3.2). The calculated area of overlap are summarized in Table A1.1. Parameters μ, *N, h,* appear to have minimal effect on the overlaps. Higher *k* leads to lower overlap between the linked and unlinked cases. Lower *c* increases the overlaps in general.

Table A3.1: The area of overlap between the distributions of π under the following three different linkage assumptions: linked (*a*=0.01, *b*=0.1), Wright-Fisher (only 1 trait), and unlinked (*a*=0).

	Main simulation	μ=0.001	*c*=0.8	*N*=200	*h*=8	*k*=9	*N*=200, *h*=8, *k*=2	*N*=500, *h*=9, *k*=16
linked & Wright-Fisher	0.61	0.63	0.90	0.66	0.60	0.44	0.6	0.44
linked & unlinked	0.25	0.26	0.42	0.28	0.18	0.11	0.24	0.04

Hitchhiking is, as in the main text, more likely to occur when there are more links (higher *a* and lower *b*)(Fig. A3.3) for all other values of the parameters. In most cases, as the link frequency approaches 1, the probability reaches 0.9-1. Where the copy rate is lower (*c*=0.8), the probability of hitchhiking driving the associative variant above 0.5 becomes very low even if link frequency is high.

Regarding how long the associated variant can remain at high frequency, again the result is qualitatively similar to those presented in the main text. The parameters *a* and *b* do not have a clear effect on how long the neutral associated variant can maintain a high frequency (Fig. A3.4, only calculated when >10% simulations are available).

Figure A3.1: Pairwise difference under unbiased transmission with different *μ*, *k*, *N*, *c*, and *h*. The horizontal line indicates the Wright-Fisher model under the corresponding *μ*, *k*, and *N* values. With the exception of panel C, the qualitative results are identical to the one shown in the main text (panel A). When there are no links or when the links are rare, $\pi_1 > \pi^{WF}$, when links are common, $\pi_1 = \pi^{WF}$, and when the links are at an intermediate frequency and *b* is small, $\pi_1 < \pi^{WF}$. We run 100 simulations for each parameter combinations.

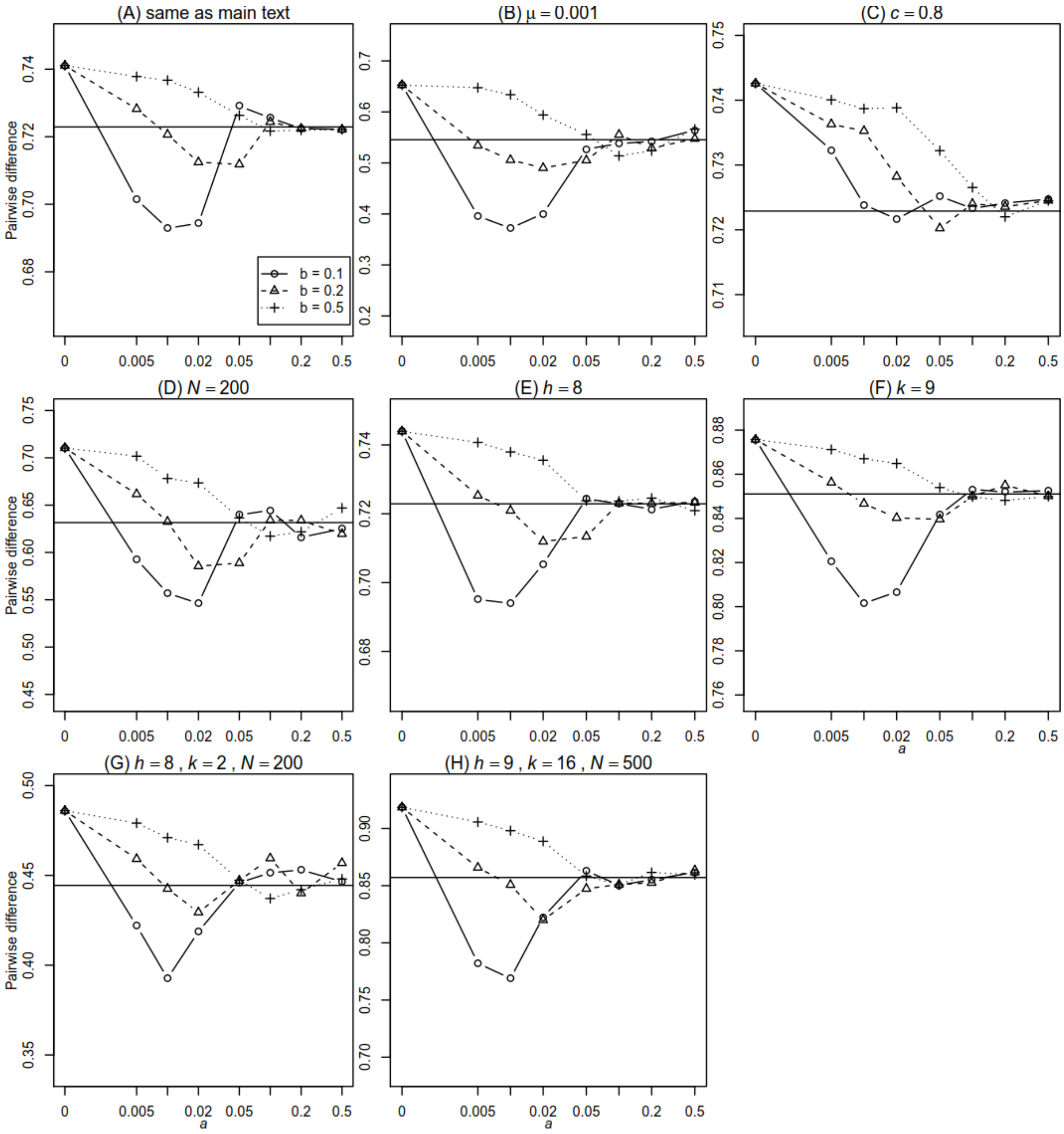

Figure A3.2: Overlap in the distributions of pairwise difference. The relative positions of the distributions remain similar and the area of overlap can again be calculated. Black lines in panels B-H represents 100 simulations while all other lines represent 1000 simulations.

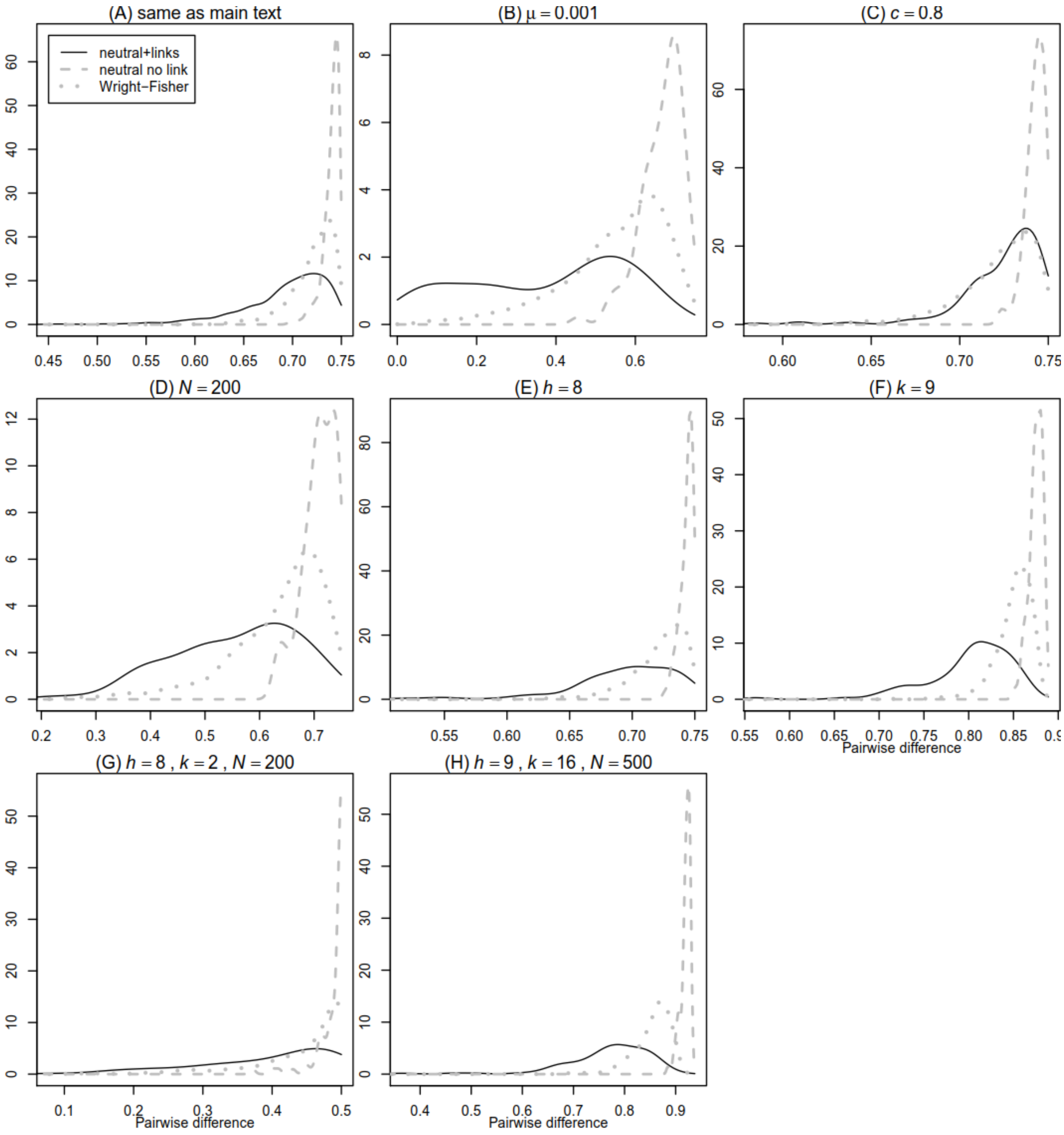

Figure A3.3: The proportion of simulations in which a neutral variant (trait 2 variant *k*) reached frequency of 0.5 by hitchhiking. Like the result presented in the main text (panel A), with more links (higher *a* and lower *b*), hitchhiking is more likely to occur. The only parameter that drastically affect hitchhiking is *c*. In panel A each data point represents 700 simulations, while in panels B-F each data point represents 100 simulations.

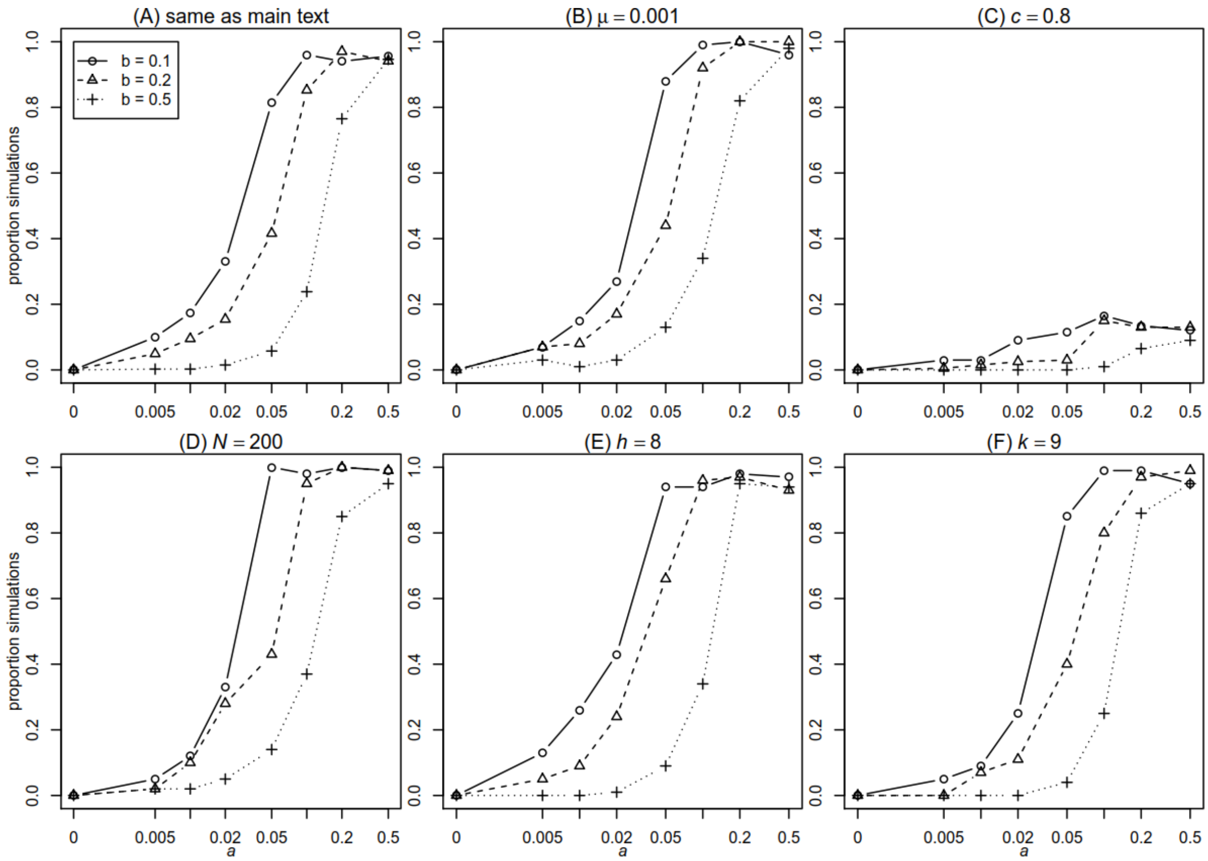

Figure A3.4: The number of timesteps in which the neutral associated variant (trait 2 variant *k*) can stay at high frequency. Like the result presented in the main text (panel A), the parameters *a* and *b* do not have a clear effect on how long the neutral associated variant can maintain a high frequency. Only calculated if hitchhiking occurred in more than 10% of the simulation.

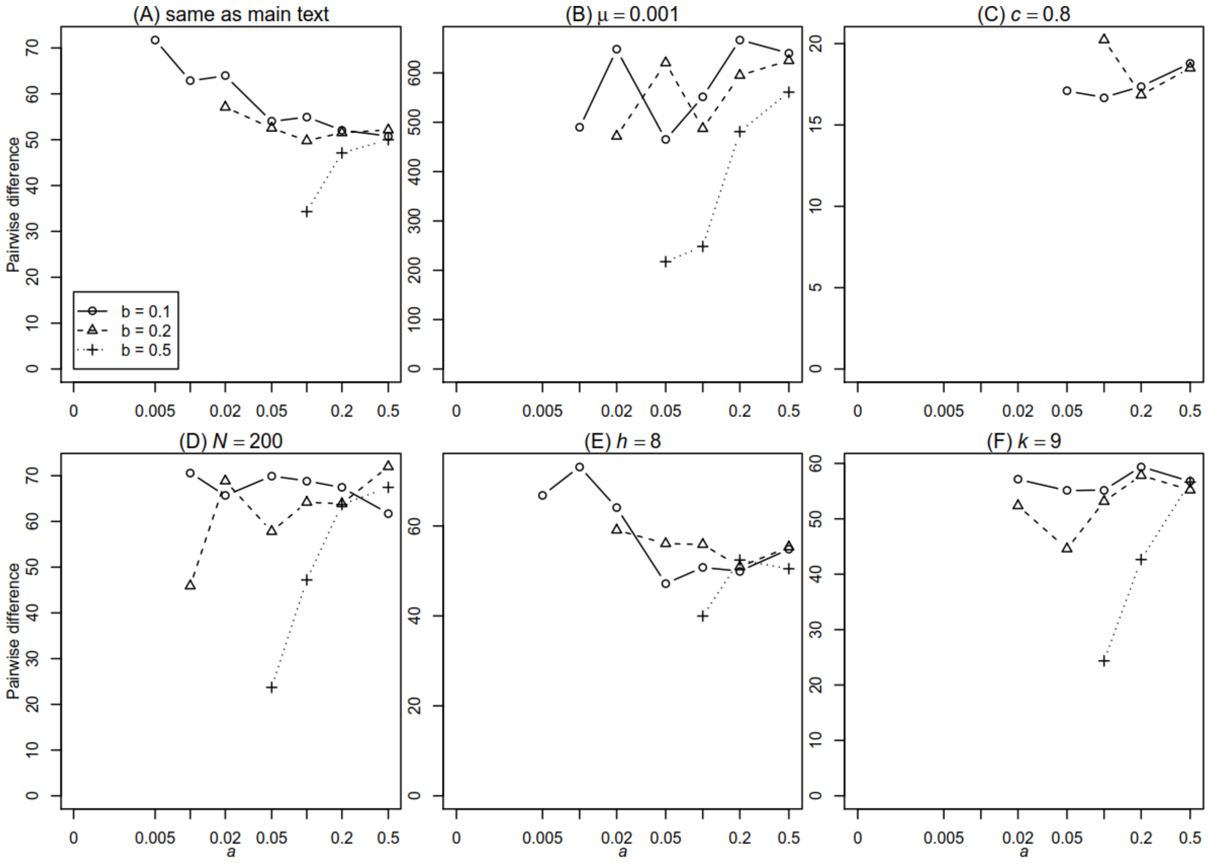

S4. Rate of adaptation

After we made the adjustments for hitchhiking at the 5000th timestep (see main text under *Simulation setup*), we also measure the average number of timesteps that occurs before the frequency of trait 1 variant 1 decreases for the first time, a sign that the selective sweep has ended. If this number is small, it suggests the rate of adaptation is high.

We find that with more links, the rate of adaptation become higher (Fig. A4.1). This is because under payoff-biased transmission, transmission increases the frequency of high-payoff variants in the population, and links increase the number of traits being transmitted in each timestep.

Figure A4.1: Mean duration of the cultural sweep. With more links, the frequency of the innovation (trait 1 variant 1) reaches its peak in shorter time. This is because more traits are transmitted, which favours the spread of the innovation due to payoff-biased transmission. (s_1=0.9, s_2=0)

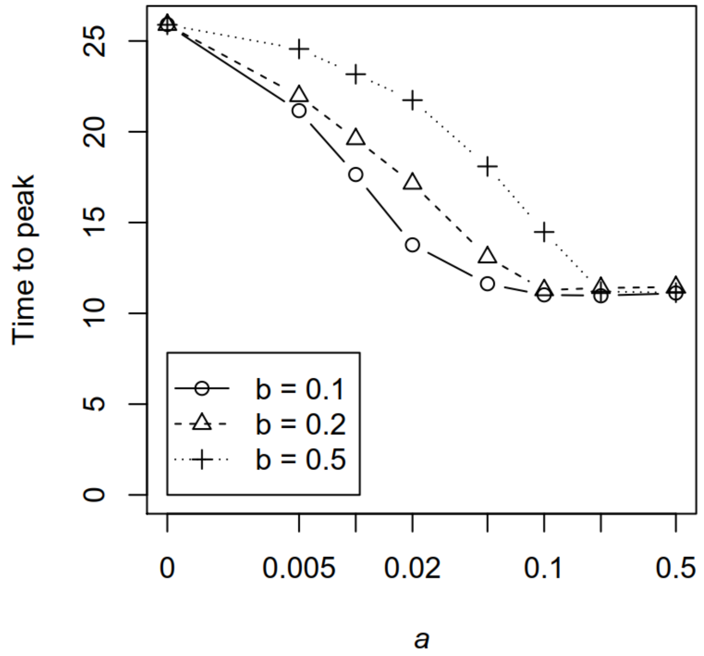

Supplement: Appendix [file rspb20191951supp1.pdf]
